# Aging Effect on Push-Out Bond Strength of Six Resin Cements: An In Vitro Study

**DOI:** 10.3390/ma18061371

**Published:** 2025-03-20

**Authors:** Eugenia Baena, Nuria Escribano, Victoria Fuentes, Isabel Reche, Laura Ceballos

**Affiliations:** 1 Sciences Faculty, Alfonso X El Sabio University, 28691 Madrid, Spain; eugenia.baena@urjc.es (E.B.); irechmar@uax.es (I.R.); 2 Health Sciences Faculty, IDIBO Research Group, Universidad Rey Juan Carlos, 28922 Madrid, Spain; victoria.fuentes@urjc.es (V.F.); laura.ceballos@urjc.es (L.C.)

**Keywords:** resin cement, bond strength, push-out test, cementation, fiber posts, aging

## Abstract

The number of resin cements marketed for fiber post cementation has increased significantly. This study compared the push-out bond strength (PBS) of self-adhesive and universal resin cements used to lute fiber posts at 24 h and after 6 months of aging in artificial saliva. Fiber posts were luted to eighty human roots endodontically treated with four self-adhesive/one-step resin cements, with one of them also used in combination with its appropriate tooth primer; one universal resin cement, applied as one-step or together with its corresponding universal adhesive (multi-step); and one adhesive/multi-step resin cement, as a control. After storage (24 h or 6 months), the interfaces were subjected to PBS tests and the data were analyzed by two-way ANOVA and Tukey and Student’s *t*-tests (*p* < 0.05 defined as statistical significance). The results showed that Scotchbond Universal Plus + RelyX Universal attained statistically higher values at 24 h and 6 months. At 24 h, all resin cements yielded similar PBS to root dentin, while at 6 months, NormoCem obtained the lowest PBS. Storage for 6 months significantly decreased PBS for NormoCem and Multilink Automix. Root section did not influence PBS regardless of storage time. It was concluded that PBS is resin cement dependent. The universal resin cement, RelyX Universal, applied in combination with Scotchbond Universal Plus adhesive, obtained a higher and more stable PBS than the other resin cements tested.

## 1. Introduction

According to the Global Burden of Disease 2021 Study, there are more than 2240 million untreated caries lesions in permanent teeth worldwide [1]. In our daily practice, many of these caries lesions are extensive and only detected when pulpal symptoms are irreversible; thus, an endodontic treatment might be needed prior to restoration. Moreover, in cases with relevant coronal structure loss, the placement of a post to retain a core restoration may be required, assuming the post does not compensate for the absence of a ferrule effect [2,3,4,5,6]. Specifically, anterior teeth and premolars would benefit more from their placement compared to molars, increasing tooth survival [7]. In this context, fiber posts are the preferred choice for dentists among all types available on the market [8,9,10]. They are characterized by a dentin-like elastic modulus and their chemical composition allows them to be adhesively bonded to root dentin, providing a better occlusal force distribution along the root canal that mitigates the risk of root fracture [11,12,13]. Furthermore, fiber posts are partially translucent, improving esthetics and the degree of conversion of resin cements used to lute these posts [14,15].

There is a wide range of resin cements available on the market and a novel simplified classification has been suggested for them as adhesive/multi-step, self-adhesive/one-step, and universal cements (one- or multi-step) [16]. Adhesive/multi-step resin cements were the first to be launched and have shown excellent clinical behavior in the long term [17,18]. Specifically, dual-cure adhesive/multi-step cements have been indicated to lute fiber posts to achieve an adequate degree of conversion where light cannot reach [19,20]. However, the previous application of an adhesive system makes them sensitive to technique and operator [21]. Therefore, self-adhesive/one-step resin cements have become the first choice for dentists to lute fiber posts as their clinical use is simpler while attaining a similar bond strength to adhesive/multi-step cements [22,23]. These cements are all dual-cure and composed of acidic functional and hydrophilic monomers that simultaneously demineralize and infiltrate the substrate, resembling that of mild self-etching adhesives in their adhesion mechanism to dentin [24,25]. Therefore, since these cements adhere directly to dentin, they do not require the use of a primer or adhesive, minimizing the operator’s influence and reducing application time [26,27]. Accordingly, post debonding has been considered the main reason for failure of teeth restored with fiber posts [28]. 

In the last few years, a novel group of resin cements have been developed, the universal resin cements. Their philosophy is that these cements can be used to lute any type of bonded restoration, applying them either in self-adhesive mode (one-step) or after an adhesive application (multi-step) [16]. If the multi-step option is chosen, a compatible universal adhesive must be applied in self-etch or etch-and-rinse mode prior to the cement application to provide improved bonding effectiveness [29,30]. This versatility is very attractive for clinicians because it reduces to one the number of resin cements that are needed to solve clinical situations in which adhesive requirements are different. Moreover, although they contain photo-initiators and redox initiators to enable a dual cure, some of them also include a “touch-cure” or “contact-cure” polymerization when used together with their appropriate universal adhesive [31,32], resembling some dual-cure adhesive/multi-step resin cements, to increase their self-curing activity [33], which is specially required for fiber post luting.

As the clinical success of post-core restorations is considered dependent on a stable retention of fiber posts to root dentin [34,35], the selection of the resin cement resin is of paramount importance. Nevertheless, information regarding the performance of universal resin cements on fiber post luting, in comparison with other resin cements, as well as regarding the need of a combined application with their respective universal adhesives, is limited [36].

Therefore, the aim of this in vitro study was to investigate the immediate and 6 months push-out bond strength along the root canal length of four self-adhesive/one-step resin cements, one of them also used in combination with its appropriate tooth primer; one universal resin cement, applied as one-step or together with its corresponding universal adhesive (multi-step); and one adhesive/multi-step resin cement. Thus, the present investigation tested the following null hypothesis: the retention of FRC posts is not influenced by the resin cement used, the root section, or the storage time.

## 2. Materials and Methods

Eighty single-rooted human teeth (maxillary central incisors and mandibular premolars [37]) extracted for periodontal or orthodontic reasons were used in the present study, and were stored in 0.1% thymol (Sigma-Aldrich, St. Louis, MO, USA) at 4 °C for a period no longer than 6 months after being extracted. Teeth with fully closed apices were selected, without caries or restorations, no previous endodontic treatment, with a root curvature less than 10 degrees and without root resorption. After debris removal, roots were perpendicularly sectioned at the enamel cementum junction.

The sample size calculation was based on a previous study [38] evaluating the push-out bond strength of posts luted with RelyX Unicem and G-Cem at two different root levels. The sample size was estimated at a 95% confidence interval and power level of 80%, which resulted in a minimum of 4 samples per experimental group.

### 2.1. Root Canal Treatment

Working length was established at 1 mm less than the total length of the root canal. Then, a glide path was prepared with Proglider and Protaper Gold system files, systematically used according to the manufacturer’s instructions (Dentsply Sirona, Ballaigues, Switzerland). The final working file was F2 or F3 depending on the canal dimensions. Patency was kept between the files with a 10 manual file and 5.25% sodium hypochlorite irrigation. A final irrigation was performed with distilled water and 17% EDTA for 1 min (Ultradent, South Jordan, UT, USA). The canals were dried with calibrated paper points and obturated with gutta-percha (Dentsply Sirona) and a resin endodontic sealer (AH-Plus, De Trey, Zurich, Switzerland) with a vertical thermoplastic obturation technique (System B Elements Free, Sybron Dental Specialties, Orange, CA, USA).

### 2.2. Post Space Preparation

Once the endodontic treatment was performed, the post space was prepared at 9 mm leaving a minimum apical seal of 4 mm. Gutta-percha was removed using Gates–Glidden burs size 2 and 3 (Dentsply Sirona) and then refrigerated drills for size 0 or 1 posts were used according to the canal size (FRC Postec Plus Intro Pack, Ivoclar Vivadent, Schaan, Liechtenstein). The selected fiber posts were FRC Postec Plus (Ivoclar Vivadent), previously cut at 13 mm to leave 4 mm out of the canal. The post space was cleaned with distilled water and dried. According to the manufacturer’s instructions, the fiber posts were etched with phosphoric acid for 60 s, then washed and dried. The silane Monobond-S (Ivoclar Vivadent) was applied for 60 s and carefully dried.

Afterward, 5 roots were randomly distributed to each of the 8 experimental groups according to the resin cement used for post luting and the mode of application, following the manufacturers’ instructions (Table 1):

RX: Self-adhesive/one-step resin cement RelyX Unicem 2 (Solventum, St. Paul, MN, USA).

GC: Self-adhesive/one-step resin cement G-Cem (GC Europe, Luzern, Switzerland).

NC: Self-adhesive/one-step resin cement Normocem (Normon, Tres Cantos, Spain).

RU: Universal/one-step (self-adhesive mode) resin cement RelyX Universal (Solventum).

SBUp-RU: Universal adhesive Scotchbond Universal Plus (Solventum) was applied and photopolymerized for 10 s, prior to the universal/multi-step (self-etch mode) resin cement RelyX Universal.

GO: Self-adhesive/one-step resin cement G-Cem One.

AEP-GO: G-Cem One Adhesive Enhancing Primer (GC Europe) application followed by self-adhesive/multi-step resin cement G-Cem One.

MA (Control group): Self-etch primer Multilink Primer (Ivoclar Vivadent) was applied before the adhesive/multi-step resin cement Multilink Automix.

**Figure 1:**
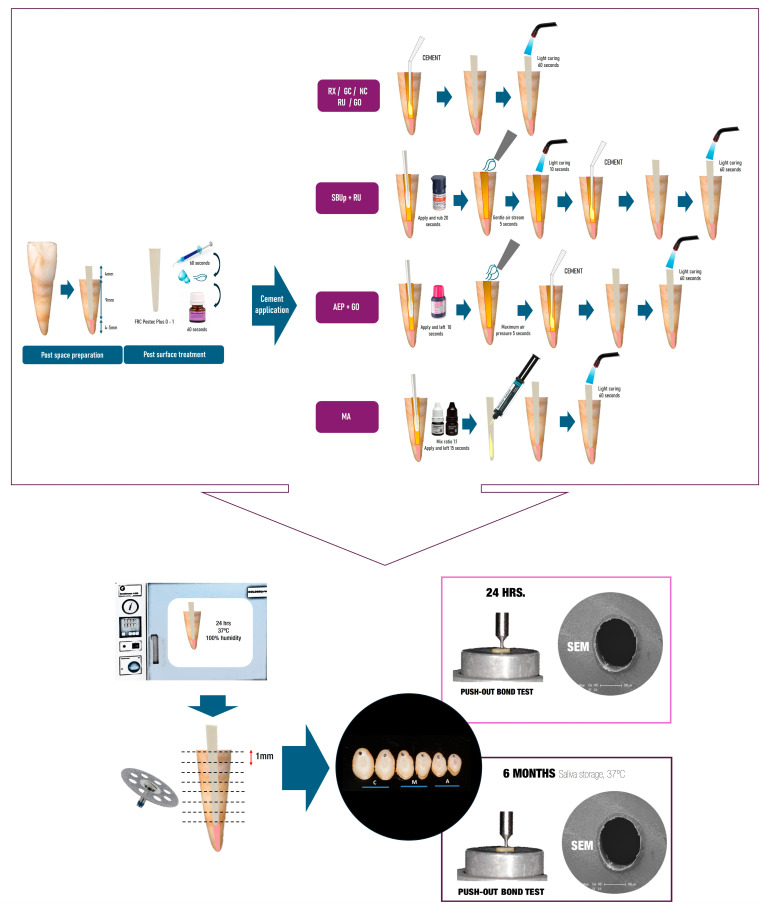
Schematic representation of the post cementation procedure in each experimental group and the SBS and SEM tests for each evaluation time.

All resin cements were light-cured through the post for 60 s (Spec3, Coltène, Alstätten, Switzerland) with the light tip in contact with the post upper side. Afterward, all specimens were stored for 24 h at 37 °C and 100% humidity. Figure 1 illustrates the preparation and evaluation procedures for the experimental groups.

### 2.3. Specimen Preparation

Roots were transversally sectioned along the radicular length into 6 slices 1 mm thick, two corresponding to each of the root thirds, using a slow-speed diamond saw under refrigeration (Isomet 5000, Buehler, Lake Bluff, IL, USA).

Half of the slices were immediately subjected to push-out bond strength testing and the other half were stored for 6 months in artificial saliva, replaced every two weeks, at 37 °C and 100% humidity. All slices were photographed under a stereomicroscope (Olympus, SZX7, Hamburg, Germany) at ×30 magnification to measure the coronal and apical radii of the fiber post. The obtained images were analyzed with ImageJ software (v. 1.44, National Institutes of Health, Bethesda, MD, USA).

### 2.4. Push-Out Test

The slices were fixed face down with cyanoacrylate (Loctite Gel, Henkel, Düsseldorf, Germany) on the push-out base. Fiber posts were submitted to push-out bond strength testing at a crosshead speed of 0.5 mm/min (Instron 3345, Instron Corp., Canton, MA, USA). The adhesive bond strength was calculated in megapascals (MPa) by dividing the debonding force registered in Newtons (N) by the interfacial area (A, mm^2^) of the post calculated using the truncated cone formula: A = π(R + r)[h^2^ + (R − r)^2^] × 0.5, where π = 3.14, R = fiber post coronal radius, r = fiber post apical radius, and h = slice thickness.

### 2.5. Failure Mode

After push-out testing, debonded specimens were inspected under a stereomicroscope (Olympus, SZX7) to classify the type of failure as adhesive (between dentin/resin cement or post/resin cement), cohesive (within the post, resin cement, or tooth) or mixed (adhesive and cohesive simultaneously). Pre-test failures that occurred during cutting or afterward were registered. Representative specimens from each experimental group were gold sputtered and analyzed under SEM (Phillips XL30 ESEM, FEI Company, Hillsboro, OR, USA) at ×100 and ×350 magnification.

### 2.6. Statistical Analysis

The mean and standard deviations of push-out bond strength (PBS) values were calculated for each experimental group. As the results met normality and homoscedasticity conditions (Shapiro–Wilk and Levene tests, respectively), three-way ANOVA was performed to evaluate the influence of the independent variables (resin cement, root section, and storage time) on the push-out bond strength. Post hoc comparisons were performed using Tukey and Student’s *t*-tests for independent variables. Additionally, the distribution of the types of failure was analyzed by Chi-square tests. The significance level was set at *p* < 0.05 for all statistical tests. All statistical tests were performed using IBM SPSS Version 27.0 (IBM, Armonk, NY, USA).

## 3. Results

### 3.1. Push-Out Test

The mean PBS values and standard deviations registered for each experimental group at each root section and storage time are shown in Table 2.

Three-way ANOVA showed that PBS values were influenced by the resin cement (*p* < 0.001), storage time (*p* = 0.013), the interaction between storage time and resin cement (*p* < 0.001), and storage time and root third (*p* = 0.025). The root section and its interaction with resin cement had no influence on PBS (*p* > 0.05). Finally, the interaction among the three variables (resin cement, storage time and root section) was also not statistically significant (*p* > 0.05).

Therefore, the adhesive strength values of each resin cement were compared without distinguishing the root section for each storage time. At 24 h, similar PBS values were determined for all resin cements tested, except for the universal cement SBUp-RU in self-etch mode, which attained significantly higher mean values (22.2 MPa). After 6 months of storage, posts bonded with the same universal adhesive and resin cement combination again showed the highest values (22.8 MPa). The lowest mean PBS values were determined for NC (3.3 MPa), without statistical differences compared to MA (7.3 MPa). The rest of the resin cements evaluated attained intermediate and similar bond strength values, ranging from 11.5 MPa for the self-adhesive RU to 17.5 MPa for GO used in self-etch mode.

The influence of root section on PBS was statistically significant at 24 h (*p* = 0.04), but not at 6 months (*p* > 0.05). At baseline, and for all resin cements, PBS values in the apical third were lower than in the middle third and similar to those obtained in the coronal third.

Regarding the influence of storage time on bond strength, only posts bonded with MA and NC exhibited a significant decrease after 6 months of saliva storage (*p* < 0.001).

### 3.2. Failure Mode Analysis

**Figure 2:**
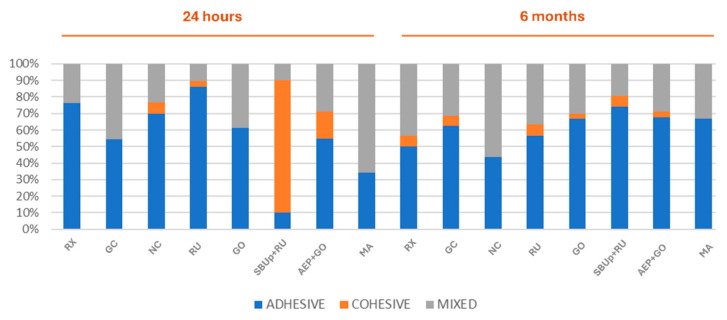
Type of failure distribution (%) for each experimental group at 24 h and 6 months.

The failure mode distribution is shown in Figure 2. At 24 h, significant differences in the failure modes among the resin cements were detected (*p* < 0.001). Adhesive failure between the resin cement and dentin was the most representative for almost all resin cements, except for SBUp-RU (mainly cohesive failures) and MA (mainly mixed failures). At 6 months, all groups showed a similar distribution of failure mode (*p* > 0.05). Adhesive failures between resin cement and dentin were the most predominant, except in the cases in which the resin cements RU and GO were applied with their respective adhesive or primer, as failures were detected at the resin cement–post interface. Moreover, cohesive failures were scarce in comparison with 24 h.

**Figure 3:**
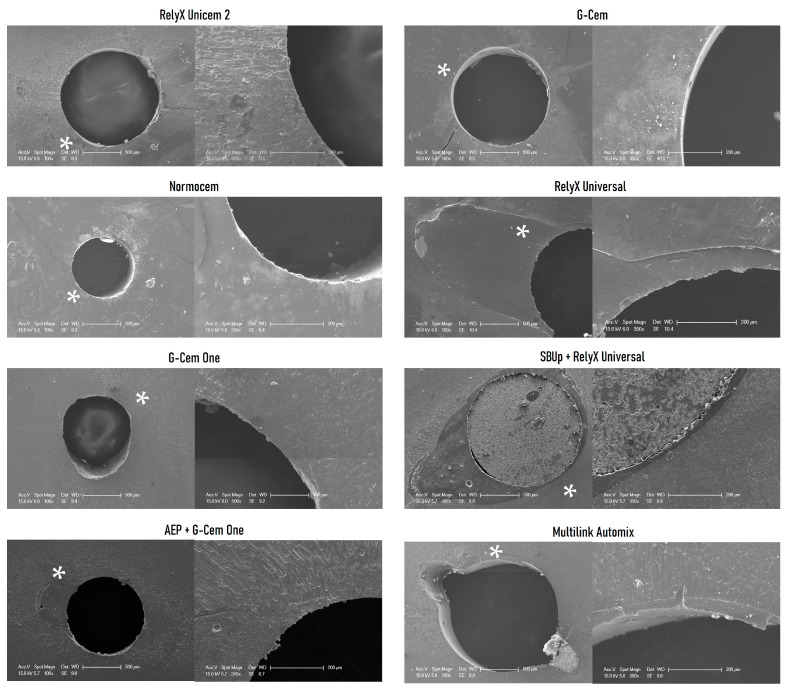
SEM images of representative push-out failures at 24 h, general view and interface detail (×100/×350 magnification). The white asterisk indicates the selected area magnified at ×350. RX, GC, NC, and GO images show typical adhesive failures at the dentin–resin cement interfaces without root canal walls devoid of resin cement remnants. The SUBp-RU image represents an adhesive failure at the resin cement–post interface without a complete debonding of the fiber post, which is still attached to the post space and the adhesive and the resin cement remain bonded to the root dentin. The AEP-GO and RU images show adhesive failures at the resin cement–post interface and the presence of cement around the dentin walls. The MA image exhibits a mixed failure with remnants of the resin cement still attached to the dentin walls.

**Figure 4:**
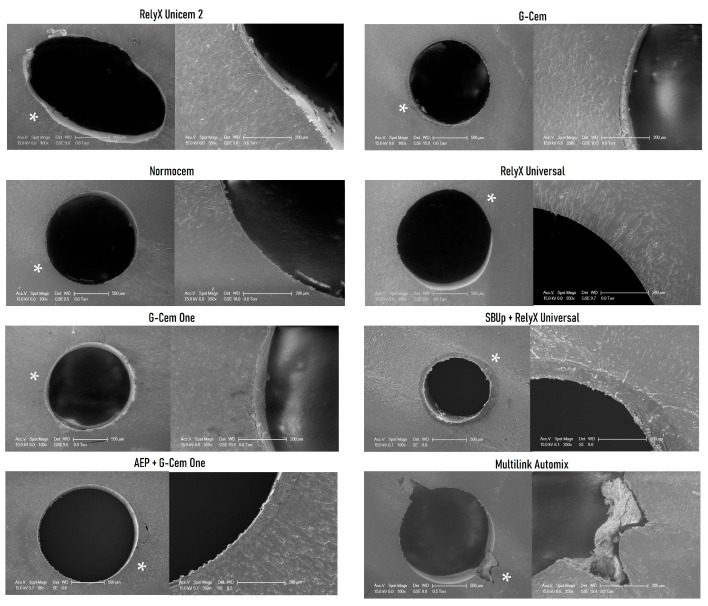
SEM images of representative push-out failures at 6 months, general view and interface detail (×100/×350 magnification). The white asterisk indicates the selected area magnified at ×350. RX, GC, NC, RU, GO and MA images predominantly show areas free of cement that correspond to adhesive failures at the dentin–resin cement interface, with occasional remnants that may be due to the slightly oval section of the canal. SUBp-RU and AEP-GO images exhibit adhesive failures at the resin cement–post interface where the resin cement is still attached to the dentin walls after the PBS test.

SEM images of representative failures for the resin cements tested at both storage times are displayed in Figure 3 (24 h) and Figure 4 (6 months).

## 4. Discussion

In the present study, the null hypothesis tested was rejected as the resin cement used and the storage time significantly affected push-out bond strength values, as well as the interaction between resin cement and storage time, and between storage time and root section.

The push-out test has been widely used to evaluate resin cement bond strength within the root canal, and it is a valid method to simulate fiber post clinical failures because the load is applied parallel to the bonded interface and different root regions can be evaluated [39,40,41]. Bonding to the root canal space is always a challenge due to insufficient light transmission to the middle and apical thirds, a high C-factor, the unavoidable presence of voids produced during luting and the presence of residues (as observed for MA in Figure 4) [42,43], which may reduce the post bond strength and, consequently, lead to post debonding [35,44]. According to Özcan and Volpato [45], a meticulous adhesive resin cementation of posts could overcome the problems of luting to the radicular dentin. Therefore, in the present study, all luting procedures were performed by the same experienced operator to minimize operator sensitivity. Moreover, all resin cements were applied using an elongation tip, which helped minimize the access difficulty to the post space [46,47].

The results obtained in the present study showed that the type of resin cement and its mode of application significantly influenced the push-out bond strength at both storage times. The highest values, as well as an increased frequency of cohesive failures, were registered when the universal resin cement RU was applied after its appropriate universal adhesive Scotchbond Universal Plus, both after 24 h and 6 months of aging.

Several circumstances may have contributed to these results. One of them is the ability of SBU to achieve effective and stable adhesion to dentin tissue, as described in previous research [48]. The presence of 10-MDP monomer in its composition is a key factor due to its high etching capability and ability to chemically bond to calcium from hydroxyapatite, forming MDP–calcium salts [49,50]. These salts generate a strong and stable bond that is highly resistant to degradation [51]. It is particularly interesting that this occurred despite the presence of a smear layer as it was applied in self-etch mode [52]. Other researchers have also reported an improved bond performance of this universal resin cement when used not as a self-adhesive one-step, but as a multi-step [53,54]. Additionally, the universal adhesive was photopolymerized before resin cement insertion, unlike AEP that was co-polymerized with GO, obtaining a higher immediate bond strength [55]. Of course, the previous photopolymerization of the adhesive requires its careful application and adequate air-thinning as the fit of the post could be impaired, especially in the apical third. Nevertheless, SBUp-RU engages in “touch-cure” polymerization when both come into contact with an effectiveness that allows for the self-cure of both without a detrimental effect on bond strength [54].

One of the self-adhesive resin cements tested in our study, G-Cem One, is marketed with an optional Adhesive Enhancing Primer (AEP) for higher bond strength demands that also provides the touch-cure polymerization feature. In contrast, fiber posts luted with G-Cem One did not benefit from AEP application in terms of improved immediate or delayed PBS, despite containing functional acidic monomers such as 10-MDP and 4-MET. Other authors have reported an increased PBS of prefabricated fiber posts with AEP application [33]. According to Ozaki et al. [56], the adhesive primer had a mild but lasting effect on the cement degree of conversion during the initial polymerization phase (30–60 min.), contributing to an adequate initial hardness that would enhance the final mechanical properties.

Nonetheless, the only noticeable change when the AEP was applied was the type of failure, as adhesive failures were mainly located between the post and the cement, rather than at the cement–dentin interface. Moreover, there was an increase in cohesive failures at 24 h, as depicted in Figure 2 and SEM images (Figure 3 and Figure 4). Although adhesive failures (dentin–cement interface or cement–post interface) were grouped for statistical analysis, this type of adhesive failure located at a cement–post interface was also observed when RU was used in combination with SUPp, indicating stronger bonds at dentin–cement interfaces for these combinations. In fact, it was common to obtain specimens where the post did not fully dislodge and only a displacement was observed, as shown in SEM images corresponding to 24 h for SBUp-RU.

Differences between the SBUp-RU and AEP-GO groups could arise because AEP was applied and co-polymerized with the resin cement G-Cem One according to the manufacturer’s instructions. Also, variations in composition could explain the differences between both groups. SBUp contains camphorquinone (CQ) combined with a tertiary amine to boost chemical polymerization. AEP contains an alternative initiator, diphenyl (2,4,6-trimethylbenzoyl) phosphine oxide, known as TPO, which showed an advantage over CQ regarding polymerization efficiency and water compatibility [57]. However, one of TPO’s disadvantages may be its different light-absorption spectrum (380–425 nm) versus that of CQ/amine (470 nm), needing a broader spectrum source of light [58], which is difficult to achieve when curing through a fiber post [59]. A previous study [60] revealed significantly higher bond strength values for CQ/Amine than for TPO-containing adhesive formulations, which is in accordance with our results.

The rest of the resin cements tested obtained a similar PBS to GO, with or without AEP, at 24 h, while after 6 months of aging in artificial saliva, NC and MA demonstrated statistically lower values.

In general, self-adhesive resin cements contain two types of monomers: methacrylates and acidic monomers with carboxylic or phosphoric acids. These acidic monomers demineralize the tissue while infiltrating it [59]. And although they have a lower demineralization ability and weaker mechanical interlocking [59] than adhesive/multi-step resin cements, RU (the predecessor of Rely X Unicem 2) GO has been demonstrated to be a valid alternative to lute fiber posts in clinical settings [61,62].Their mechanism of adhesion is not solely dependent on mechanical interlocking, as they also chemically interact with hydroxyapatite [63,64], explaining their acceptable bond strength along the radicular length observed in the present study. As a singularity, RU contains a redox initiator, which diffuses into the smear layer to enhance bonding to dentin and leads to the formation of a highly cross-linked three-dimensional network. This leads to higher bond strength values, thereby reducing the influence of the curing mode [54]. This reduction could explain the uniform behavior of the cement along the root canal.

The other self-adhesive resin cement tested, NC, demonstrated similar PBS values at 24 h, but it significantly decreased after the 6-month storage in artificial saliva. Due to its recent launch on the market, there is no literature available for this product. However, its susceptibility to hydrolytic degradation suggests that the monomers included in its composition may not form stable calcium salts. This trend was also observed for MA [23], which achieved good immediate PBS values, together with a high percentage of mixed failures (Figure 2). Multilink Automix requires the prior application of the self-etching Multilink Primer to demineralize the dentin and facilitate the subsequent infiltration of the resin cement. In a previous study [65] comparing the adhesive strength of four dual-cure resin cements, MA exhibited immediate PBS values similar to those obtained in our study [66]. These results were related to the high HEMA content of Automix, which can effectively fill the gap between the collagen fibers to form a mixed layer. MA bases its bonding to dentin on acidic adhesive monomers, with a 2.3 pH value. In a recent study [34], MA showed clear, thin high-density layers that may be evidence of a chemical interact6on between functional monomers and hydroxyapatite in the dentin, and it was considered a reliable luting system. Nevertheless, MA showed a reduction in PBS values over time, at 6 months. This can be due to the low resistance to hydrolysis of HEMA [67], its main functional monomer, in comparison with the stable calcium salts that self-assemble into nano-layers [68].

Regarding the effect of root section on PBS, no differences were detected among the resin cements when tested after 6 months of saliva storage. However, there were differences in the 24 h evaluation, with lower adhesion values recorded in the apical third for all cements compared to the middle and coronal thirds. The literature on this topic is controversial. Some studies suggest that PBS is influenced by the depth of the post space and the density of dentinal tubules, which is lower in the apical regions. These factors are critical for both adhesive/multi-step and self-adhesive/one-step resin cements, leading to higher bond strength values in the coronal thirds [51,69,70,71]. However, other studies [23,71] suggest that the behavior of resin cement along the radicular length depends on the specific cement tested. These studies indicate that bond strength values are similar regardless of root depth, as observed in our study at the 6 month evaluation.

The current results cannot be directly extrapolated to clinical scenarios as this is an in vitro study. Additionally, the behavior of the materials appears to be material-dependent, preventing any general conclusions about the behavior of these cements as a group. Future in vitro studies should evaluate the influence of masticatory forces on fiber posts’ bond strength in order to evaluate a situation closer to the clinical scenario. In addition, future clinical studies should emphasize the necessity of applying the appropriate adhesive before using a universal resin cement, given the favorable clinical outcomes of self-adhesive resin cements. The combination of universal resin cement and its corresponding universal adhesive may be particularly beneficial in cases with limited retention, such as when the post does not fully conform to the canal anatomy or is excessively short, thereby compromising its bond strength to dentin.

## 5. Conclusions

Within the limitations of this in vitro study, the results suggest that using the universal cement RelyX Universal after Scotchbond Universal Plus application helps to stabilize the bond strength in the long term, maintaining the high bond strength achieved immediately after fiber post cementation.

## Figures and Tables

**Table 1:** Type of resin cement, composition and mode of application according to their respective manufacturers.

Material/Manufacturer	Type of Resin Cement	Resin Matrix and Filler Type	Dentin Pretreatment	Mode of Application
RelyX Unicem 2 (RX)/Solventum	Self-adhesive One-step	Mixture of GPDMA, bisGPDMA and trisGPDMA	None	Apply with an Endo Tip in the canal. Insert fiber post. Excess cement removal. Photopolymerize.
G-Cem (GC)/GC Europe	Self-adhesive One-step	UDMA, PAE, 4-META, DM, fluoro-aluminosilicate glass, silicon dioxide	None	Apply with GC capsule elongation tip in the canal after mixing. Insert fiber post within 1 min after cement application. Excess cement removal. Photopolymerize.
Normocem (NC)/Normon	Self-adhesive One-step	Mixture of DM, glass powder, fumed silica, PAE, catalysts, stabilizer, pigments	None	Apply with an Endo-Tip in the canal. Insert fiber post. Photopolymerize.
RelyX Universal (RU)/Solventum	Universal One-step (RU)	Mixture of GPDMA, bisGPDMA and trisGPDMA, novel amphiphilic redox initiator system, radiopaque fillers	None	Apply with the elongation tip in the canal. Insert fiber post. Excess cement removal. Photopolymerize.
	Universal Multi-step (SBUp-RU)		Scotchbond Universal Plus (SBUp) Bis-GMA, 10-MDP, 2-HEMA, silanes, silica, ethanol, CQ, water, Vitrebond copolymer	Apply with disposable applicator. Rub it in for 20 s. Direct gentle air stream over it for 5 s. Photopolymerize for 10 s. Apply RU as described above
G-Cem One (GO)/GC Europe	Self-adhesive One-step	Fluoro-aluminosilicate glass, DM, initiator, stabilizer, pigment, silicon dioxide, MDP, trimethoxysilane, 6-tert-butyl-2,4-xylenol, 2,6-di-tert-butyl-p-cresol, EDTA disodium salt dehydrate, vanadyl acetylacetonate, TPO, ascorbic acid, CQ, magnesium oxide.	None	Extrude the material into the post space using an elongation tip. Insert the post within 1 min after cement application. Excess cement removal. Photopolimerize Let the material set for 4 min.
	Self-adhesive Multi-step (AEP-GO)		G-Cem One Adhesive Enhancing Primer (AEP) 10-MDP, ethanol, DM resins, butylated hydroxytoluene	Apply with disposable applicator. Leave undisturbed for 10 s. Dry for 5 s under maximum air pressure. Remove excess with paper points. Apply GO as described above. Photopolymerize.
Multilink Automix (MA)/Ivoclar Vivadent	Adhesive Muti-step	Bis-GMA, HEMA, 2-dimethylaminoethyl methacrylate, ethyoxylated bisphenol A dimethacrylate, UDMA, barium glass, ytterbium trifluoride, spheroid mixed oxide	Self-etch Multilink primer Primer A: water, initiators, sulfonate amine Primer B: HEMA, phosphonic acid acrylate, methacrylate modified polyacrylic acid, stabilizers	Mix the two Multilink Primer liquids A and B in a 1:1 mixing ratio (1 drop of each primer). Apply the mixture in the canal using a thin micro-brush. Let react for 15 s. Remove excess with paper points. Dispense MA from the double-push syringe and mix the two pastes in a 1:1 ratio on the mixing pad. Coat the post with the mixed cement and place it in the canal. Excess cement removal. Photopolymerize.

**Table 2:** Mean PBS and standard deviation (sd) in MPa for each resin cement tested, according to root third and storage time.

Experimental Groups		24 h Mean (sd)			6 Months Mean (sd)		24 h vs. 6 Months *p* Values
RX	Coronal	8.9 (5.8)	12.0 (7.2) b	Coronal	10.5 (5.2)	11.5 (5.5) cd	0.766
	Middle	15.7 (9.0)		Middle	11.8 (4.8)		
	Apical	11.4(5.4)		Apical	12.2 (6.6)		
GC	Coronal	14.3 (3.8)	12.7 (5.6) b	Coronal	13.0 (6.5)	14.2 (6.9) bc	0.350
	Middle	10.6 (6.8)		Middle	14.1 (5.5)		
	Apical	13.2(5.7)		Apical	15.3 (8.5)		
NC	Coronal	13.5 (7.0)	13.4 (6.8) b	Coronal	4.4 (4.2)	3.3 (2.9) e	<0.001
	Middle	11.6 (2.6)		Middle	2.6 (1.9)		
	Apical	15.2 (9.2)		Apical	2.9 (2.3)		
RU	Coronal	13.7 (4.2)	13.0 (4.5) b	Coronal	10.5 (8.7)	12.3 (6.0) c	0.615
	Middle	14.7 (3.2)		Middle	14.5 (4.4)		
	Apical	10.3(5.1)		Apical	11.9 (3.3)		
GO	Coronal	14.5 (4.0)	13.0 (6.9) b	Coronal	14.2(8.0)	15.2 (7.7) bc	0.259
	Middle	15.8 (9.0)		Middle	15.6 (9.1)		
	Apical	9.2 (5.5)		Apical	15.6 (6.5)		
SBUp-RU	Coronal	22.3 (9.9)	22.2 (10.1) a	Coronal	19.5 (5.1)	22.8 (7.0) a	0.789
	Middle	27.0 (9.2)		Middle	23.3 (6.9)		
	Apical	17.2 (9.5)		Apical	25.3 (7.8)		
AEP-GO	Coronal	16.5 (5.2)	15.0 (5.5) b	Coronal	16.6 (4.9)	17.5 (8.5) b	0.182
	Middle	14.4 (3.5)		Middle	15.7 (7.6)		
	Apical	14.2 (7.3)		Apical	19.9 (11.6)		
MA	Coronal	15.1 (5.1)	13.8 (5.0) b	Coronal	8.6 (5.2)	7.3 (4.6) de	<0.001
	Middle	14.6 (3.3)		Middle	7.4(4.1)		
	Apical	12.2 (5.9)		Apical	5.9 (4.3)		

Same lower-case letters mean statistically similar PBS among experimental groups at 24 h and after 6 months of storage.

## Data Availability

The original contributions presented in this study are included in the article. Further inquiries can be directed to the corresponding author.
